# Trust first, concerns second: An international vignette study of older adults' preferences towards deprescribing statins

**DOI:** 10.1002/bcp.70440

**Published:** 2026-01-13

**Authors:** Sarah E. Vordenberg, Noelia Dulo, Carissa Bonner, Eliza Ferguson, Vincent D. Marshall, Kristie Rebecca Weir

**Affiliations:** ^1^ College of Pharmacy University of Michigan Ann Arbor Michigan USA; ^2^ School of Public Health University of Michigan Ann Arbor Michigan USA; ^3^ Faculty of Medicine and Health, Sydney School of Public Health, Leeder Centre for Health Policy, Economics, and Data University of Sydney Sydney New South Wales Australia; ^4^ Faculty of Medicine and Health, Sydney School of Public Health University of Sydney Sydney New South Wales Australia; ^5^ Institute of Primary Health Care (BIHAM) University of Bern Bern Switzerland

**Keywords:** content analysis, deprescribing, international, medicine, survey

## Abstract

This study investigated the attitudes and beliefs of older adults towards deprescribing statins in Australia, the United Kingdom and the United States, using an online, vignette‐based study. Presented with a hypothetical scenario in which a general practitioner advised stopping simvastatin, participants rated their level of agreement and explained their rationale. Analysis was conducted incorporating the Patient Deprescribing Typology (PDT), which asks participants to share medication‐related learning style, beliefs about importance, decision‐making preferences and attitudes towards deprescribing. The findings correlated with participants' personal experiences with statins and their willingness to deprescribe in the scenario. The results highlight the importance of adapting deprescribing decisions to patients' beliefs and backgrounds to support shared decision‐making. Future research is needed to assess whether typology‐based screening tools can improve patient‐centred deprescribing conversations in clinical practice.

**What is already known about this subject**

- Approximately one‐half of older adults take unnecessary or inappropriate medicine leading to functional decline, cognitive impairment, adverse drug events and increased health‐care costs.
- Understanding of the specific reasons older adults agree or hesitate to deprescribe can inform tailored deprescribing communication strategies.

**What this study adds**

- The Patient Deprescribing Typology provides a framework for understanding what matters to older adults when they consider deprescribing.
- Trust in health‐care providers was the leading factor in older adults' statin deprescribing decisions (63.9%), followed by medicine concerns (40.7%), while patient‐specific factors and beliefs about continuation were less common.

## 1 INTRODUCTION

Many older adults are prescribed unnecessary or potentially harmful medicines.^1^, ^2^ Deprescribing, the process of discontinuing medicine when the risks outweigh the benefits, is a medicine optimization strategy.^3^ This approach requires careful consideration of an individual's health status, functional capacity, goals, and personal preferences.^3^ While the importance of engaging older adults in shared decision‐making about their medicines is widely acknowledged,^4^ implementing deprescribing in clinical practice remains challenging.

Older adults have diverse and nuanced medicine perspectives, including some who are hesitant to discontinue medicines, even when advised by their health‐care provider.^5^, ^6^ This led members of the study team to develop the Patient Deprescribing Typology (PDT) that categorizes individuals based on their attitudes towards medicines, decision‐making preferences and openness to deprescribing. These findings suggest the need for tailored communication strategies that align with patients' perspectives, but it remains unclear what matters most to patients within each typology and how they navigate deprescribing decisions.

This study builds on a prior experimental survey^7^ that (1) used hypothetical vignettes to quantitatively assess older adults' willingness to discontinue an HMG‐CoA reductase inhibitor (‘statin’) and (2) was subsequently analysed via a latent class analysis, which revealed four PDT classes.^8^ Here, we present findings from a subsequent content analysis, which examines themes associated with the PDT classes.

## 2 METHODS

### 2.1 Study design and administration

This study was part of a larger vignette‐based online experiment.^7^ Participants were aged 65 years and older and lived in Australia (AU), the United Kingdom (UK), and the United States (US). Qualtrics Research Panels (Provo, UT) recruited participants from 9 August to 20 September 2021. Sampling quotas were used to ensure roughly equal representation by gender with a total target of 1200 participants per country, based on previous research.

The participants read a vignette about ‘Mrs. EF’ a 76‐year‐old patient taking 11 medicines to manage multiple health conditions. Her General Practitioner (GP) recommended stopping simvastatin (control). The primary outcome was agreement with the deprescribing recommendation: ‘I think that Mrs. EF should follow her GP's recommendation and stop taking the simvastatin’ on a 6‐point Likert scale, with ‘*Strongly disagree* (1)’ and ‘*Strongly agree* (6)’ as the scale anchors. An optional free‐text response box was provided, and the participants explained why they selected the response.

We asked participants four medicine‐related questions aligned with the PDT, a framework grounded in qualitative research identifying three core patient types.^9^, ^10^ A previously published latent class analysis of the same dataset identified four distinct classes, providing a quantitative, exploratory extension of the typology.^8^ The participants reported the number of medicines they currently take, support they receive to manage their medicines and experience taking a statin. We collected demographic characteristics, including age, country of residence, gender and education. Finally, we asked the participants to report their self‐reported health and level of confidence in filling out medical forms.^11^, ^12^, ^13^

### 2.2 Content analysis

We conducted a content analysis in which qualitative data are analysed by themes and overarching domains.^14^ All data except the comments provided by the participants were hidden throughout the coding process, which took place in Excel, version 2302 (MicroSoft Corporation). The initial coding framework was developed based on the content of the vignette and our previous content analyses.^6^, ^15^ Additional codes were generated based on the contextual factors and themes observed while reading a sample of the responses. The coding framework was revised iteratively and piloted by K.R.W., N.D. and S.V. The same random set of responses (*n* = 50) was coded independently by two investigators (K.R.W. and N.D.), with high interrater agreement (Cohen *κ* = 0.8). Discussions between coauthors resolved conflicts. After N.D. coded all responses, 20% were double‐coded (K.R.W.) with high agreement (*κ* = 0.8). The full coding framework included 21 codes (including miscellaneous). We consolidated two similar themes, resulting in the ‘distrust in recommendation’ theme. A total of 19 themes are reported, including four domains and four subdomains created based on thematic content.

### 2.3 Statistical analysis

We previously conducted a latent class analysis of the four PDT questions, which identified four types of older adults related to deprescribing within this dataset: Class 1 ‘Trusts their doctor’, Class 2 ‘Makes own decisions’, Class 3 ‘Avoids deprescribing’ and Class 4 ‘Medicine are not important’.^8^ This study builds on that work by analysing participants' free‐text responses to explore how reasons for agreeing or disagreeing with deprescribing varied across these classes. Descriptive analysis was used to assess the frequency and percentage of each theme, subdomain and domain by participant. We also compared the latent classes by using participants' propensity to agree (score 4–6) or disagree (score 1–3) with deprescribing and experience with taking a statin, and the test was the odds ratio, with a Bonferroni adjustment for the family‐wise error rate. We compared the deprescribing strata by using Fisher's exact test. We used a statistical significance level of two‐sided *P* < .05. Statistical analysis was performed with R statistical software (v. 4.3.1).^16^ The study was registered at http://ClinicalTrials.gov Identifier: NCT04676282 and was deemed exempt by the University of Michigan Institutional Review Board (HUM00183129).

## 3 RESULTS

The original study included 4873 participants. We excluded participants from the Netherlands (*n* = 1204) as their responses were in Dutch. Because of missing data from a programming error, we also excluded the group of participants who were asked to make a decision for themselves (*n* = 1613) rather than the hypothetical patient ‘Mrs. EF’. We excluded participants with responses that were two words or less in length (*n* = 357), participants with missing data (*n* = 122) or participants whose data could not be matched between the original latent class analysis and content analysis datasets (*n* = 11).

### 3.1 Participant characteristics

Approximately one‐third of participants (*n* = 1566) resided in each country (Table 1). The participants were female (*n* = 824, 52.6%) and had a median age of 70 years (interquartile range 68–74 years). The participants reported having a high school diploma or less (*n* = 493, 31.5%), or attending trade school, some college, or having an associate's degree (*n* = 518, 33.1%). Approximately one‐half of the participants (*n* = 776, 49.6%) reported currently taking a statin.

**TABLE 1:** Demographic characteristics, clinical experiences and attitudes towards stopping medicine in a hypothetical vignette (*n* = 1566).

	Number (percent)
Demographic characteristics	
Age in years, median (interquartile range)	70 (68–74)
Country	
Australia	522 (33.3)
United Kingdom	543 (34.7)
United States	501 (32.0)
Gender	
Male	742 (47.4)
Female	824 (52.6)
Education level	
High school diploma or less	493 (31.5)
Trade school, some college, or associate's degree	518 (33.1)
Bachelor's degree	363 (23.2)
Master's degree or higher	192 (12.3)
Health status^a^	
Excellent	80 (5.1)
Very good	344 (22.0)
Good	653 (41.7)
Fair	417 (26.7)
Poor	71 (4.5)
Confidence filling out medical forms (health literacy)	
Extremely	869 (55.5)
Quite a bit	480 (30.7)
Somewhat	134 (8.6)
A little bit	50 (3.2)
Not at all	33 (2.1)
Medicine experiences	
Support to manage medicine^a^	
Minimal	1373 (88.4)
Occasional	129 (8.3)
Complete	52 (3.4)
Personal experience with HMG‐CoA reductase inhibitor (statin)	
Never	663 (42.3)
In the past	127 (8.1)
Current	776 (49.6)
Number of medicines, median (interquartile range)	5 (3–8)
Prescription, median (interquartile range)	3 (2–6)
Over‐the‐counter medicines and supplements, median (interquartile range)	1 (0–3)
Medicine decision‐making preferences	
Patient deprescribing typology	
Class 1 – ‘Trusts their doctor’	752 (48.0)
Class 2 – ‘Makes own decisions’	407 (26.0)
Class 3 – ‘Avoids deprescribing’	265 (16.9)
Class 4 – ‘Medicines are not important’	142 (9.1)
Hypothetical vignette	
Agreement with stopping statin in hypothetical vignette	
1 – *Strongly disagree*	68 (4.3)
2	102 (6.5)
3	154 (9.8)
4	278 (17.8)
5	493 (31.5)
6 – *Strongly agree*	471 (30.1)

^a^ Sum less than 1566 because of missing responses.

### 3.2 Domains

In the content analysis, the participants most frequently expressed the ‘Trust Domain’ (*n* = 1001, 63.9%) (Table 2). The participants who shared positive attitudes and beliefs, such as a desire to follow the doctor's advice because of their high levels of trust, were classified as having ‘Trust in Medical Expertise’ (*n* = 801, 51.2%). In contrast, some participants (*n* = 242, 15.5%) shared comments that were classified into the ‘Questioning Expertise Subdomain’, such as due to being doubtful of the doctor's knowledge (*n* = 167, 10.7%), needing additional information before making a deprescribing decision (*n* = 65, 4.2%), desiring a second opinion (*n* = 60, 3.8%), or reporting that they value a specialists opinion more than a general practitioner (*n* = 43, 2.8%).

**TABLE 2:** Frequency of themes provided by participants by domain and patient deprescribing typology class.

Domain *Subdomain*	Theme	Description	Representative quote	Total number of participants (%)	Class 1 ‘Trusts their doctor’ (*n* = 752)	Class 2 ‘Makes own decisions’ (*n* = 407)	Class 3 ‘Avoids deprescribing’ (*n* = 265)	Class 4 ‘Medicines are not important’ (*n* = 142)
**Trust and expertise of the physician (trust domain)**				**1001 (63.9)** ^a^	**547 (72.7)**	**218 (53.6)**	**141 (53.2)**	**95 (66.9)**
*Positive trust and expertise subdomain*				801 (51.2)^b^	492 (65.4)	165 (40.5)	62 (23.4)	82 (57.7)
	Trust in medical expertise	Trust in the competence and advice of doctors.	*‘I trust my doctor completely and follow any advice he gives me.’*	801 (51.2)	492 (65.4)	165 (40.5)	62 (23.4)	82 (57.7)
*Questioning expertise subdomain*				*242 (15.5)*	*78 (10.4)*	*62 (15.2)*	*87 (32.8)*	*15 (10.6)*
	Distrust in recommendation	Distrust or doubt in the GP's knowledge or recommendations.	*‘I disagree with the GP as I have had doctors who don't know the patient well enough.’*	167 (10.7)	47 (6.2)	37 (9.1)	75 (28.3)	8 (5.6)
	Information needs	Requiring additional data or context to make decisions.	*‘I need more information… what are Ms. EF's cardiac risk factors?’*	65 (4.2)	21 (2.8)	24 (5.9)	14 (5.3)	6 (4.2)
	Second opinion	Seeking a second opinion to confirm or challenge the primary recommendation.	*‘I would have gotten a second opinion before discontinuing medication.’*	60 (3.8)	22 (2.9)	18 (4.4)	18 (6.8)	2 (1.4)
	Specialist authority	Trust in specialists' opinions over general practitioners.	*‘I think she should see her cardiologist before she stops.’*	43 (2.8)	11 (1.5)	8 (2.0)	22 (8.3)	2 (1.4)
**Perceived medicine necessity and risks (medicine domain)**				**637 (40.7)**	**259 (34.4)**	**230 (56.5)**	**90 (34.0)**	**58 (40.8)**
*Medicine concerns subdomain*				*464 (29.6)*	*207 (27.5)*	*175 (43.0)*	*35 (13.2)*	*47 (33.1)*
	Harms and side effects	Concerns about adverse effects of the medicine.	*‘The side effects could cause the dizziness she is experiencing.’*	279 (17.8)	123 (16.4)	109 (26.8)	18 (6.8)	29 (20.4)
	Unnecessary medicine	Belief that the medicine is no longer needed.	*‘If there is no need to take it, it is reasonable to stop it.’*	209 (13.4)	98 (13.0)	77 (18.9)	13 (4.9)	21 (14.8)
	Polypharmacy burden	Concerns about taking multiple medicine.	*‘She is taking too many drugs that may react with each other.’*	84 (5.4)	32 (4.3)	33 (8.1)	9 (3.4)	10 (7.0)
*Balancing options subdomain*				*361 (23.1)*	*135 (18.0)*	*128 (31.4)*	*71 (26.8)*	*27 (19.0)*
	Risk *vs*. benefits	Weighing the potential benefits against the harms.	*‘The benefits are outweighed by the possible side effects.’*	214 (13.7)	87 (11.6)	73 (17.9)	36 (13.6)	18 (12.7)
	Exploring alternatives	Considering other medicines or options.	*‘Maybe switch to atorvastatin.’*	159 (10.2)	52 (6.9)	60 (14.7)	38 (14.3)	9 (6.3)
**Patient‐specific factors (patient domain)**				**295 (18.8)**	**114 (15.2)**	**99 (24.3)**	**60 (22.6)**	**22 (15.5)**
	Ageing	Adjusting medicines owing to ageing and changing health.	*‘Over time the body changes, and medicines have to adapt.’*	111 (7.1)	48 (6.4)	43 (10.6)	14 (5.3)	6 (4.2)
	Long‐term use	Impact of medicine use over time.	*‘The earlier prescription was beneficial; now it is less so.’*	76 (4.9)	37 (4.9)	23 (5.7)	7 (2.6)	9 (6.3)
	Personal experience	Response informed by personal or family experience.	*‘I have known a relative that had a problem with simvastatin side effects.’*	62 (4.0)	25 (3.3)	14 (3.4)	18 (6.8)	5 (3.5)
	Social context	Considering social and family responsibilities.	*‘Given her husband's stroke, I would be wary of doing the same.’*	44 (2.8)	9 (1.2)	12 (2.9)	21 (7.9)	2 (1.4)
	Lifestyle changes	Emphasis on diet and exercise as alternatives.	*‘She should be advised on diet and appropriate exercise.’*	33 (2.1)	8 (1.1)	16 (3.9)	7 (2.6)	2 (1.4)
**Beliefs supporting continuation (continuing domain)**				**233 (14.9)**	**57 (7.6)**	**47 (11.5)**	**117 (44.2)**	**12 (8.5)**
	Prevention	Focus on preventing future health issues.	*‘Stopping may increase levels of cholesterol and risk of diseases.’*	129 (8.3)	36 (4.8)	29 (7.1)	55 (20.8)	9 (6.3)
	Health consequences	Fear of negative health outcomes from stopping medicine.	*‘The danger of having a stroke after stopping that medication now at her age is too dangerous.’*	128 (8.2)	29 (3.9)	23 (5.7)	70 (26.4)	6 (4.2)
	Positive perceptions	Belief that the medicine is effective.	*‘Simvastatin helps keep her cholesterol under control.’*	80 (5.1)	19 (2.5)	13 (3.2)	45 (17.0)	3 (2.1)
	No problems with medicine	Belief that the medicine is harmless or beneficial.	*‘Why stop taking it if it isn’t hurting her?’*	35 (2.2)	10 (1.3)	6 (1.5)	18 (6.8)	1 (0.7)

^a^ The domain reflects the number (percentage) of participants with one or more themes within the domain.

^b^ The subdomain reflects the number (percentage) of participants with one or more themes within the subdomain.

The ‘Medicine Domain’ (*n* = 637, 40.7%) included the ‘Medicine Concerns Subdomain’ (*n* = 464, 29.6%) and ‘Balancing Options Subdomain’ (*n* = 361, 23.1%). Concerns raised by participants included harms and side effects from the current medicine (*n* = 279, 17.8%), taking an unnecessary medicine (*n* = 209, 13.4%), or issues such as drug interactions that can occur when taking multiple medicines (*n* = 84, 5.4%). The ‘Balancing Options Subdomain’ included participants considering the risks and benefits of taking or stopping the medicine (*n* = 214, 13.7%) and a preference to explore alternatives, such as a different medicine (*n* = 159, 10.2%).

The participants less frequently shared the ‘Patient Domain’ (*n* = 295, 18.8%). Associated themes focussed on adjusting medicines because of physiologic changes associated with ageing (*n* = 111, 7.1%), decreasing benefits of the medicine over time (*n* = 76, 4.9%), personal experience with this medicine (*n* = 62, 4.0%), considerations related to the social context (*n* = 44, 2.8%) and a preference for lifestyle changes (*n* = 33, 2.1%).

The ‘Continuing Domain’ (*n* = 233, 14.9%) included themes related to a desire to continue the medicine to prevent future health issues (*n* = 129, 8.3%), fear of negative consequences if the medicine were stopped (*n* = 128, 8.2%), beliefs that the medicine is effective (*n* = 80, 5.1%) and a desire to continue the medicine because it is not causing harm (*n* = 35, 2.2%).

### 3.3 Patient deprescribing typology classes

In the latent class analysis, the participants were most frequently assigned to Class 1 ‘Trusts their doctor’ (*n* = 752, 48.0%). These participants frequently shared comments that aligned with the ‘Trust Domain’ because of their high level of trust in the doctor's expertise (*n* = 492, 65.4%). In contrast, the participants assigned to Class 2 ‘Makes own decisions’ (*n* = 407, 26.0%) frequently shared comments aligning with the ‘Medicine Domain’ (*n* = 230, 56.5%), including related to the ‘Medicines Concerns Subdomain’ (*n* = 175, 43.0%) and ‘Patient Domain’ (*n* = 99, 24.3%). The participants assigned to Class 3 ‘Avoids deprescribing’ (*n* = 265, 16.9%) frequently reported comments associated with the ‘Continuing Domain’ (*n* = 117, 44.2%). Finally, the participants assigned to Class 4 ‘Medicines are not important’ (*n* = 142, 9.1%) frequently reported the ‘Trust Domain’ (*n* = 95, 66.9%) and the ‘Medicine Domain’ (*n* = 58, 40.8%).

**FIGURE 1:**
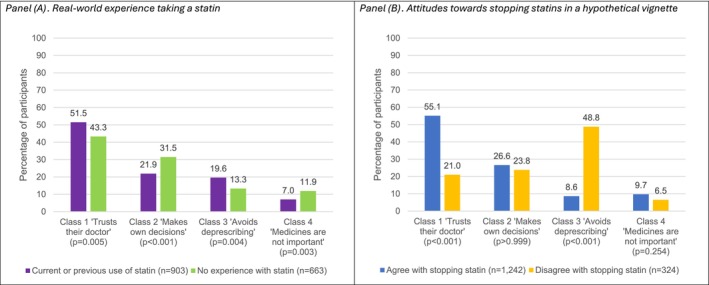
Patient deprescribing typology by (A) experience and (B) attitudes with HMG‐CoA reductase inhibitors (statins).

We also evaluated class assignments by personal experience taking statins and attitudes towards stopping them within a hypothetical vignette (Figure 1). The participants with current or previous experience taking a statin most frequently were assigned to Class 1 ‘Trusts their doctor’ (*n* = 465, 51.5%) and least likely to be assigned to Class 4 ‘Medicines are not important’ (*n* = 63, 7.0%) (Figure 1A). This same pattern was observed among participants without experience taking a statin. Furthermore, the participants who agreed with stopping a statin in the hypothetical vignette were most frequently assigned to Class 1 ‘Trusts their doctor’ (*n* = 684, 55.1%), while those who preferred to continue the statin were most often assigned to Class 3 ‘Avoids deprescribing’ (*n* = 158, 48.8%) (Figure 1B). Appendix A presents a full comparison of participants who agreed and disagreed with the recommendation to stop the statin in the hypothetical vignette, categorized by the Patient Deprescribing Typology.

## 4 DISCUSSION

In this study of older adults in three countries, most participants expressed strong trust in their doctors' expertise, with over half conveying a willingness to follow medical advice because of this trust. However, the participants also frequently cited concerns about the benefits and potential harms of stopping a statin. Latent class analysis revealed four distinct classes of participants, with personal statin experience and deprescribing preferences in a hypothetical vignette aligning with the class assignments. The PDT classes may provide a foundation for tailoring deprescribing communication strategies to address patient concerns and support shared decision‐making.

These findings align with our previous work, identifying participants who agreed with a deprescribing recommendation were interested in following the doctor's recommendation because of their expertise, as well as the lack of benefit or potential harm from continuing the medicine.^15^ We also observed similarities in the studies among participants who had doubts about deprescribing, such as valuing information from a specialist or a preference for a second opinion.^6^ In this study, we saw more comments about contextual factors, such as the health of a spouse, that could influence participants' perspectives, which was likely because of differences in how the surveys were constructed.^7^ Some research has shown that patient‐specific factors are key in decision‐making about medicines.^17^ However, other research has found that medicine‐related characteristics (e.g. symptom management *vs*. preventive care) influence older adults' decisions.^18^, ^19^, ^20^

This study focusses on a key area of debate in deprescribing research; the discontinuation of statins for primary prevention in older adults.^21^, ^22^, ^23^, ^24^ Studies have raised concerns about the safety of deprescribing statins,^25^, ^26^ and findings from a systematic review suggests that statin discontinuation may be associated with worse outcomes in non‐randomized studies.^27^ However, the balance between potential benefits versus harms of statin therapy, and patient preferences, particularly in those without cardiovascular disease, remains uncertain.

Patient perspectives can vary significantly. A study of 5693 older adults found 70.4% of current users believed statins were safe, compared to only 37.4% of those who discontinued and 36.9% who declined therapy (*P* < .001).^28^ Furthermore, cardiovascular disease prevention guidelines offer limited guidance on statin discontinuation in older adults with declining health. While some recommend discontinuation in cases of poor health or limited life expectancy, decisions for adults over 75 should be individualized through shared decision‐making, balancing risks, benefits and patient preferences owing to the lack of high‐quality evidence.^29^, ^30^

In this study, we observed variations in the frequencies with which older adults were assigned to the PDT classes based on their personal experience with statins. Therefore, we plan to conduct a follow‐up study exploring how older adults respond to a medicine‐specific PDT. In this dataset, we observed four classes; however, subsequent research identified three classes in a 14‐country survey of 1131 older adults (≥65 years, ≥5 medications) recruited by general practitioners,^31^ broadly consistent with the original qualitative work^9^ and later quantitative exploration.^10^ Furthermore, based on our findings, we are testing a two‐item questionnaire that allows older adults to share who they would like to be involved in making medication decision (e.g. doctor, self, family member, friend or caregiver) and their preference towards deprescribing current medications (yes, maybe, no). Additional research is needed to determine whether incorporating these two questions into the electronic health record could facilitate patient‐centred deprescribing conversations during routine clinical practice.

### 4.1 Strengths and limitations

The primary strength of our study is that data were collected from three countries. Utilizing an online survey allowed us to efficiently collect data about patient attitudes, which is important as deprescribing interventional studies infrequently capture patient‐reported preferences.^32^ In addition, older adults have reported enjoying experimental surveys.^33^ Additionally, our findings replicate and extend a previous study with a different participant group, providing further support for older adults' preferences and beliefs in the context of deprescribing. Finally, the researchers coded the free‐text responses without access to other variables to maintain blinding. The free‐text response was optional to ensure that participants did not feel compelled to respond; however, most participants decided to share their opinion.

We acknowledge that content analysis includes subjective interpretation of data, and decisions made as part of a hypothetical vignette may not fully capture real‐world decisions. Furthermore, although efforts were made to recruit a diverse group of respondents, achieving a fully representative sample was not possible, as participants were those who had access (e.g. reliable Internet), were able (e.g. sufficient health literacy and health status) and willing (e.g. interested in research) to engage in an online survey. Finally, we may not have captured all potential themes, as we excluded participants with missing data caused by a coding error, free‐text responses of two words or fewer, and responses from participants in the Netherlands because they were provided in Dutch.

## AUTHOR CONTRIBUTIONS

Sarah Vordenberg and Kristie Weir contributed to the study's conceptualization and design.

All authors participated in data acquisition, analysis or interpretation. Kristie Weir and Sarah Vordenberg wrote the first draft of the manuscript, with all authors contributing to revisions and approving the final version. Eliza Ferguson and Noelia Dulo provided administrative and technical support. Vince Marshall conducted the statistical analyses. Carissa Bonner advised on interpretation and revising the manuscript. Sarah Vordenberg secured funding for the study, and she and Kristie Weir supervised the research. Both Sarah Vordenberg and Kristie Weir had full access to the study data and serve as guarantors.

## CONFLICT OF INTEREST STATEMENT

We have no conflicts of interest to declare.

## Data Availability

The data that support the findings of this study are available upon reasonable request from the corresponding author. Some data may not be publicly available because of privacy or ethical restrictions.

## Appendix A Comparison of participants who agreed and disagreed with the deprescribing recommendation by Patient Deprescribing Typology

**Table:**

	Class 1			Class 2			Class 3			Class 4		
	‘Trusts their doctor’			‘Makes own decisions’			‘Avoids deprescribing’			‘Medicines are not important’		
	Number of participants (%)		p‐value	Number of participants (%)		p‐value	Number of participants (%)		p‐value	Number of participants (%)		p‐value
Domain	Agree (*n* = 684, 91.0%)	Disagree (*n* = 68, 9.0%))		Agree (*n* = 330, 81.1%)	Disagree (*n* = 77, 18.9%)		Agree (*n* = 107, 40.4%)	Disagree (*n* = 158, 59.6%)		Agree (*n* = 121, 85.2%)	Disagree (*n* = 21, 14.8%)	
*Subdomain*												
Theme												
Trust and expertise of the physician (trust domain)	510 (74.6)	37 (54.4)	**0.001**	182 (55.2)	36 (46.8)	0.205	77 (72.0)	64 (40.5)	**<0.001**	79 (65.3)	16 (76.2%)	0.453
*Trust in medical expertise subdomain*	483 (70.6)	9 (13.2)	**<0.001**	160 (48.5)	5 (6.5)	**<0.001**	59 (55.1)	3 (1.9)	**<0.001**	77 (63.6)	5 (23.8%)	**0.001**
Trust in medical expertise	483 (70.6)	9 (13.2)	**<0.001**	160 (48.5)	5 (6.5)	**<0.001**	59 (55.1)	3 (1.9)	**<0.001**	77 (63.6)	5 (23.8%)	**0.001**
*Questioning expertise subdomain*	49 (7.2)	29 (42.6)	**<0.001**	29 (8.8)	33 (42.9)	**<0.001**	26 (24.3)	61 (38.6)	**0.017**	4 (3.3)	11 (52.4%)	**<0.001**
Distrust in recommendation	27 (3.9)	20 (29.4)	**<0.001**	13 (3.9)	24 (31.2)	**<0.001**	17 (15.9)	58 (36.7)	**<0.001**	1 (0.8)	7 (33.3%)	**<0.001**
Information needs	16 (2.3)	5 (7.4)	**0.034**	14 (4.2)	10 (13.0)	**0.007**	7 (6.5)	7 (4.4)	0.577	2 (1.7)	4 (19.0%)	**0.004**
Second opinion	14 (2.0)	8 (11.8)	**<0.001**	10 (3.0)	8 (10.4)	**0.010**	8 (7.5)	10 (6.3)	0.805	1 (0.8)	1 (4.8%)	0.275
Specialist authority	4 (0.6)	7 (10.3)	**<0.001**	2 (0.6)	6 (7.8)	**0.001**	5 (4.7)	17 (10.8)	0.111	0 (0.0)	2 (9.5%)	**0.021**
Perceived medicine necessity and risk (medicine domain)	236 (34.5)	23 (33.8)	>0.999	197 (59.7)	33 (42.9)	**0.010**	39 (36.4)	51 (32.3)	0.510	55 (45.5)	3 (14.3%)	**0.008**
*Medicine concerns subdomain*	198 (28.9)	9 (13.2)	**0.004**	164 (49.7)	11 (14.3)	**<0.001**	24 (22.4)	11 (7.0)	**<0.001**	47 (38.8)	0 (0.0%)	**<0.001**
Harms and side effects	117 (17.1)	6 (8.8)	0.086	99 (30.0)	10 (13.0)	**0.002**	10 (9.3)	8 (5.1)	0.215	29 (24.0%)	0 (0.0%)	**0.008**
Unnecessary medicine	94 (13.7)	4 (5.9)	0.087	76 (23.0)	1 (1.3)	**<0.001**	12 (11.2)	1 (0.6)	**<0.001**	21 (17.4)	0 (0.0%)	**0.043**
Polypharmacy burden	31 (4.5)	1 (1.5)	0.349	32 (9.7)	1 (1.3)	**0.010**	6 (5.6)	3 (1.9)	0.164	10 (8.3)	0 (0.0%)	0.358
*Balancing options subdomain*	116 (17.0)	19 (27.9)	**0.031**	102 (30.9)	26 (33.8)	0.683	25 (23.4)	46 (29.1)	0.325	24 (19.8)	3 (14.3%)	0.765
Risk *vs*. benefits	73 (10.7)	14 (20.6)	**0.026**	60 (18.2)	13 (16.9)	0.870	11 (10.3)	25 (15.8)	0.208	18 (14.9)	0 (0.0%)	0.075
Exploring alternatives	47 (6.9)	5 (7.4)	0.803	44 (13.3)	16 (20.8)	0.109	15 (14.0)	23 (14.6)	>0.999	6 (5.0)	3 (14.3%)	0.130
Patient‐specific factors (patient domain)	97 (14.2)	17 (25.0)	**0.031**	79 (23.9)	20 (26.0)	0.768	9 (8.4)	51 (32.3)	**<0.001**	18 (14.9)	4 (19.0%)	0.743
Ageing	40 (5.8)	8 (11.8)	0.067	39 (11.8)	4 (5.2)	0.101	4 (3.7)	10 (6.3)	0.414	6 (5.0)	0 (0.0%)	0.592
Long‐term use	33 (4.8)	4 (5.9)	0.766	18 (5.5)	5 (6.5)	0.783	0 (0.0)	7 (4.4)	**0.044**	8 (6.6)	1 (4.8%)	>0.999
Personal experience	19 (2.8)	6 (8.8)	**0.019**	11 (3.3)	3 (3.9)	0.734	3 (2.8)	15 (9.5)	**0.045**	4 (3.3)	1 (4.8%)	0.556
Social context	6 (0.9)	3 (4.4)	**0.040**	4 (1.2)	8 (10.4)	**<0.001**	2 (1.9)	19 (12.0)	**0.002**	0 (0.0)	2 (9.5%)	**0.021**
Lifestyle changes	8 (1.2)	0 (0)	>0.999	14 (4.2)	2 (2.6)	0.747	0 (0.0)	7 (4.4)	**0.044**	2 (1.7)	0 (0.0%)	>0.999
Beliefs supporting continuation (continuing domain)	29 (4.2)	28 (41.2)	**<0.001**	20 (6.1)	27 (35.1)	**<0.001**	19 (17.8)	98 (62.0)	**<0.001**	5 (4.1)	7 (33.3%)	**<0.001**
Prevention	17 (2.5)	19 (27.9)	**<0.001**	16 (4.8)	13 (16.9)	**0.001**	8 (7.5)	47 (29.7)	**<0.001**	5 (4.1)	4 (19.0%)	**0.028**
Health consequences	12 (1.8)	17 (25.0)	**<0.001**	6 (1.8)	17 (22.1)	**<0.001**	9 (8.4)	61 (38.6)	**<0.001**	2 (1.7)	4 (19.0%)	**0.004**
Positive perceptions	6 (0.9)	13 (19.1)	**<0.001**	4 (1.2)	9 (11.7)	**<0.001**	3 (2.8)	42 (26.6)	**<0.001**	0 (0.0)	3 (14.3%)	**0.003**
No problems with medicine	4 (0.6)	6 (8.8)	**<0.001**	1 (0.3)	5 (6.5)	**0.001**	4 (3.7)	14 (8.9)	0.137	0 (0.0)	1 (4.8%)	0.148
